# PCSK9‐Mediated Inflammation in Foam Cell Formation and Exploring the Biologically Active Compounds Derived From Natural Resources

**DOI:** 10.1155/adpp/3005975

**Published:** 2026-08-29

**Authors:** Maharani Hestu Mukti Wisesa, Anisa Qisti Mathriul, Vicko Suswidiantoro, Meidi Utami Puteri, Mitsuyasu Kato, Muhd Hanis Md Idris, Fadlina Chany Saputri

**Affiliations:** ^1^ Faculty of Pharmacy, Universitas Indonesia, Kota Depok 16424, Indonesia, ui.ac.id; ^2^ Laboratory of Pharmacology and Toxicology, Pharmacy Department, Faculty of Health, Universitas Aisyah Pringsewu, Lampung 35372, Indonesia, aisyahuniversity.ac.id; ^3^ Laboratory of Pharmacology, Faculty of Pharmacy, Universitas Indonesia, Kota Depok 16424, Indonesia, ui.ac.id; ^4^ National Metabolomics Collaborative Research Centre, Faculty of Pharmacy, Universitas Indonesia, Kota Depok 16424, Indonesia, ui.ac.id; ^5^ Department of Experimental Pathology, Faculty of Medicine, University of Tsukuba, Tsukuba 305-8575, Ibaraki, Japan, tsukuba.ac.jp; ^6^ Center of Drug Discovery, Integrative Pharmacogenomics Institute (iPROMISE), Universiti Teknologi MARA (UiTM) Selangor, Puncak Alam Campus, 42300, Bandar Puncak Alam, Selangor, Malaysia; ^7^ School of Biology, Faculty of Applied Sciences (FSG), Universiti Teknologi MARA (UiTM) Shah Alam, 40450, Shah Alam, Selangor, Malaysia

**Keywords:** atherosclerosis, foam cells, macrophage polarisation, natural bioactive compound, SPCSK9

## Abstract

Atherosclerosis develops as a persistent inflammatory condition that gradually alters the vascular structure and condition, in which the formation of macrophage‐derived foam cells plays a central role in disease initiation and progression. Although proprotein convertase subtilisin/kexin type 9 (PCSK9) is widely recognised for its involvement in low‐density lipoprotein receptor regulation and systemic lipid homeostasis, growing evidence indicates that PCSK9 also exerts a direct influence on macrophage inflammatory responses and intracellular lipid metabolism. Within atherosclerotic lesions, PCSK9 accelerates foam cell development by promoting oxidised low‐density lipoprotein uptake, impairing cholesterol efflux through ABCA1 downregulation and activating key inflammatory pathways, particularly those mediated by TLR4/NF‐κB and the NLRP3 inflammasome. This review emphasises recent findings that identify natural bioactive compounds as a novel and biologically relevant approach to counteracting PCSK9‐driven inflammatory foam cell formation. Compounds such as salvianolic acid B, curcumin, quercetin, fisetin and myricetin display broad antiatherogenic properties by concurrently regulating macrophage polarisation, limiting scavenger receptor expression, suppressing inflammasome activation and modulating PCSK9 expression or function. In contrast to monoclonal PCSK9 inhibitors, which primarily address lipid lowering, these natural agents offer integrated anti‐inflammatory and lipid‐modulatory effects at the cellular level. Collectively, the evidence discussed in this review positions PCSK9 modulation by natural compounds as an emerging therapeutic concept that links lipid dysregulation with vascular inflammation. This multitarget strategy may complement current lipid‐lowering therapies and support the development of more accessible and inflammation‐focused interventions for atherosclerosis.

## 1. Introduction

Atherosclerosis, one of the principal drivers of cardiovascular disease globally, develops as a consequence of sustained inflammatory activity within the arterial wall. [1]. A pivotal event in its development is the transformation of macrophages into lipid‐laden foam cells. This process is driven by the uncontrolled uptake of modified lipoproteins, particularly oxidised low‐density lipoprotein (oxLDL), via scavenger receptors (SRs) such as CD36, LOX‐1 and SR‐A [2]. Importantly, oxLDL uptake is not merely a passive lipid‐storage event; it actively initiates and amplifies inflammatory signalling. Atherosclerosis begins with endothelial dysfunction and subendothelial retention of lipoproteins, followed by LDL oxidation, endothelial activation, monocyte recruitment, macrophage differentiation and progressive cytokine amplification that collectively sustain vascular inflammation [3, 4]. Exposure to oxLDL promotes macrophage polarisation towards a proinflammatory M1 phenotype, marked by enhanced secretion of cytokines including tumour necrosis factor‐alpha (TNF‐α), interleukin‐1β (IL‐1β) and interleukin‐6 (IL‐6), alongside the activation of central inflammatory pathways such as nuclear factor kappa‐light‐chain‐enhancer of activated B (NF‐κB) and mitogen‐activated protein kinase (MAPK). Together, these mechanisms sustain and exacerbate the inflammatory milieu within atherosclerotic plaques [5].

Experimental and clinical findings suggest that proprotein convertase subtilisin/kexin type 9 (PCSK9) intensifies this inflammatory cascade [6]. Beyond its canonical role in regulating cholesterol homeostasis via hepatic LDL receptor (LDLR) degradation, PCSK9 directly influences macrophage biology within atherosclerotic lesions [7]. It exacerbates disease progression by enhancing oxLDL uptake, amplifying proinflammatory signalling through the Toll‐like receptor 4 (TLR4)/NF‐κB axis, impairing reverse cholesterol transport via the suppression of ATP‐binding cassette transporter A1 (ABCA1) and thereby accelerating foam cell formation [8, 9]. These pleiotropic actions position PCSK9 as a compelling therapeutic target that bridges dyslipidaemia and inflammation. Currently approved PCSK9‐targeting therapies, including monoclonal antibodies such as evolocumab and alirocumab [10], as well as siRNA‐based therapy Inclisiran, have demonstrated substantial LDL‐C lowering efficacy and may additionally contribute to the attenuation of vascular inflammation [11].

Concurrently, there is growing interest in natural bioactive compounds for their potential to mitigate these pathogenic processes. Substances such as curcumin, salvianolic acid B, fisetin and myricetin have demonstrated promising antiatherogenic effects [12–14]. Their mechanisms include modulating macrophage polarisation, suppressing key inflammatory pathways (e.g., NLRP3 inflammasome) and regulating the expression of SRs, offering a multifaceted approach to intervention.

This review specifically focuses on the mechanistic role of PCSK9 in macrophage‐mediated inflammation and foam cell formation during atherosclerosis progression, while also evaluating natural bioactive compounds that may modulate PCSK9‐associated inflammatory signalling pathways. Via linking molecular insights into disease pathogenesis with emerging natural therapeutic strategies, this study seeks to underscore novel approaches for combating atherosclerosis through the concurrent modulation of lipid homeostasis and inflammatory pathways. A literature search was conducted using PubMed, Scopus and Web of Science databases using combinations of the keywords ‘PCSK9’, ‘foam cell’, ‘macrophage polarisation’, ‘oxLDL’, ‘atherosclerosis’ and ‘bioactive compounds’. Articles published in English and relevant to inflammatory signalling and PCSK9‐mediated lipid dysregulation were prioritised.

## 2. Inflammatory Mechanism at the Foam Cell Formation Stage

Foam cell formation, an inflammation‐driven process critical to various disease developments, is characterised by macrophages extensively internalising oxLDL [15]. This uptake is an integral part of standard macrophage immune function, which also involves consuming pathogens [16]. Several receptors facilitate this process, notably TLR4, as well as SRs such as LOX‐1, CD36 and SR‐AI/II [17]. Specifically, LOX‐1, CD36 and SR‐AI/II manage the initial cholesterol influx during this phagocytic activity, essentially handling the initial oxLDL uptake. While SRs such as LOX‐1 and CD36 are widely expressed across various cell types, including endothelial cells, platelets, fibroblasts and smooth muscle cells [18–20]. SR‐A expression is predominantly restricted to the surface of macrophages and is particularly absent in endothelial cells [21].

### 2.1. Structural Architecture

**FIGURE 1:**
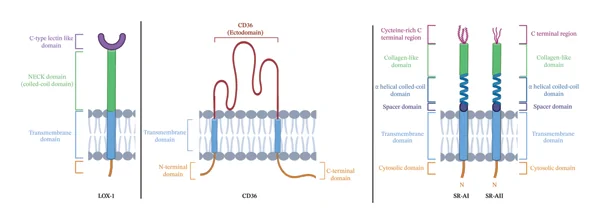
Structural domain organisation and functional comparison of LOX‐1, CD36 and SR‐A scavenger receptors involved in oxLDL recognition and foam cell formation. LOX‐1 contains a short cytoplasmic tail, transmembrane region, connecting domain, collagen‐like region and C‐type lectin‐like domain responsible for oxLDL binding. CD36 is characterised by two transmembrane domains flanking a large extracellular domain involved in the recognition of oxidised phospholipids and lipid ligands. SR‐A receptors possess cytosolic, transmembrane, extracellular spacer, collagen‐like and cysteine‐rich domains that contribute to polyanionic ligand recognition and macrophage lipid internalisation. The lower panel summarises ligand specificity, downstream signalling pathways and principal functions of each receptor in atherosclerosis progression and foam cell development. Created in BioRender. Hestu, M. (2026) https://BioRender.com/bisbrjn.

Among Class A SRs, SR‐AI and SR‐AII are the principal isoforms involved in oxLDL recognition and macrophage lipid uptake during foam cell formation [22]. SR‐A receptors possess a cytosolic domain, transmembrane region, extracellular spacer domain, collagen‐like domain and cysteine‐rich C‐terminal region. Distinctly, CD36 (SR‐B2) is a Class B SR glycoprotein defined by an extracellular domain flanked by two transmembrane domains. Finally, LOX‐1 (SR‐E1) is composed of a short N‐terminal domain, a transmembrane region, a coiled‐coil domain and a C‐type lectin‐like domain. Specifically, the C‐type lectin‐like domain of LOX‐1 harbours ten basic amino acid residues at the C‐terminus, which are critical for the binding of oxLDL. The schematic illustrates the structural architecture of three major SRs implicated in oxLDL uptake during atherogenesis: LOX‐1 (SR‐E1), CD36 (SR‐B2) and SR‐A (SR‐AI/SR‐AII) shows in Figure 1.

### 2.2. Macrophage Phenotypic Transition to M1 and the Role of Extracellular Vesicles (EVs)

Various proinflammatory cytokine agents, including IFN‐γ and oxLDL, as well as proinflammatory factors such as IL‐1β, TNF‐α and IL‐6, augment to the induction process of the macrophage phenotypic transition from type M0 (native) to M1 (proinflammatory) [23]. Shi et al. mentioned that it was proven that macrophages induced with LPS and IFN‐γ trigger the M0 to M1 transition. Both M0 and M1 macrophages release EVs, which act as fundamental mediators for intercellular communication. M0 phenotype macrophages that uptake M1‐EVs will change into M1; this is indicated by a significant increase in M1 markers, with CD86 reaching 49.93% compared to 1% in controls, and a significant 75% increase in the inducible nitric oxide synthase (iNOS) enzyme compared to other controls. Thus, when macrophages experience inflammation and have changed to M1, these cells release EVs capable of forcing other macrophages to undergo inflammation as well. M1 macrophage polarisation contributes directly to atherosclerotic progression through excessive production of proinflammatory cytokines, elevated ROS generation, amplification of chronic inflammatory signalling, extracellular matrix degradation and promotion of plaque instability [24]. The exposure of M0 macrophages to M1‐EVs activates TLR4 receptors on the cell surface, enabling the activation of inflammatory signalling pathways, such as NF‐κB, to produce various proinflammatory cytokines [25].

Upon the onset of inflammation in the tunica intima, numerous proinflammatory cytokines, including TNF‐α, facilitate the upregulation of SRA, CD36 and LOX‐1 expression [26]. Elevated nitric oxide (NO) levels are a hallmark of M1 macrophages. Upon stimulation by proinflammatory signals, activated M1 macrophages produce substantial amounts of NO through an iNOS‐mediated mechanism. Furthermore, under inflammatory conditions, the upregulation of iNOS expression drives this increased NO production [27]. A significant increase in NO production was observed in RAW264.7 cells stimulated with LPS at concentrations of 10, 50 and 100 ng/mL [28].

### 2.3. Mechanisms of oxLDL Binding and Endocytosis by Specific Receptors

The binding of oxLDL to CD36, SR‐A, LOX‐1 and TLR4 receptors triggers the initiation of endocytosis in macrophages. Specifically, CD36‐mediated endocytosis is driven by a signalling cascade involving the activation of the tyrosine kinases Lyn and Syk, which facilitates the internalisation of oxLDL particles into the macrophage cytoplasm [29]. The initiation of oxLDL endocytosis is functionally critical because excessive intracellular lipid accumulation and defective lysosomal cholesterol processing promote macrophage transformation into lipid‐laden foam cells, a hallmark event in early atherogenesis. The interaction between oxLDL and the CD36 receptor is specifically mediated by the ectodomain region. This mechanism is triggered by oxidation, which induces lipid modifications and particle shrinkage. These events disrupt the stability of β‐stranded domains, causing oxLDL to undergo a conformational transition into an amyloid‐like structure exhibiting a cross‐β‐sheet motif. This fibrillar configuration, which is homologous to β‐amyloid and apoC‐II fibrils, is bound with high affinity by macrophage CD36 and subsequently transduces a proinflammatory signalling cascade [30]. Furthermore, the CD36 receptor also recognise oxidised phospholipids (oxPC) with the key structure for high‐affinity binding is the presence of sn‐2 acyl group containing a terminal γ‐hydroxy(or oxo)‐α,β‐unsaturated carbonyl. When oxPC is transferred from oxLDL to unoxidised LDL, it also transfers the ability to induce multiple functional characteristics ascribed to oxPC [31]. Recognition of oxidised phosphatidylcholines (oxPC) by CD36 activates Src‐family kinases and downstream MAPK signalling pathways, which amplify inflammatory transcriptional responses and enhance oxLDL uptake, thereby accelerating foam cell development.

A study by Wong et al. revealed that CD36 receptors in F‐actin–poor areas were 2.6 times larger than those in F‐actin–rich ruffles. This increased receptor size facilitates the binding of large oxLDL particles, predominantly within these F‐actin–poor regions [32]. Inflammatory conditions are known to trigger the upregulation of CD36 protein expression, manifesting as an increased receptor density on the cell membrane surface. This is consistent with the findings of Gu et al., who observed elevated CD36 expression in LPS‐induced RAW 264.7 cells. This inflammatory state was characterised by decreased levels of p‐AMPKα and PGC‐1*α* compared to controls. The suppression of this signalling pathway contributes to the exacerbation of the inflammatory response, marked by a surge in the secretion of proinflammatory cytokines (IL‐1β, IL‐6, TNF‐α) and increased expression of iNOS [33].

SR‐A recognise and bind to oxLDL via its C‐terminal SR cysteine‐rich (SRCR) domains. The SR‐A variants capable of recognising acetylated or oxidised LDL include SCARA1 (SR‐A1, CD204), SCARA2, SR‐A2 and SCARA5. This recognition process is dependent on calcium ions Ca^2+^ mediated by the C‐terminal SRCR domains. Ca^2+^ serves as a critical component in SR‐A receptor recognition, facilitating coordination with Asp (aspartic acid) or Glu (glutamic acid) residues on the ligand surface. Furthermore, the SR‐A family forms homotrimers on the macrophage surface, a structural arrangement that significantly enhances ligand‐binding affinity (Cheng et al., 2021). In macrophages, JNK1 acts as an essential mediator that regulates both SR‐AI expression and oxLDL internalisation [34]. SR‐A1–mediated foam cell formation is mediated by the activation of c‐Jun N‐terminal kinase 2 (JNK2) [35].

### 2.4. Inflammatory Signalling Pathways Post‐oxLDL Binding

The interaction between oxLDL and the LOX‐1 receptor initiates proinflammatory signalling pathways involving the activation of MAPKs, particularly, p42/44 MAPK and p38 MAPK. This signal transduction subsequently culminates in the activation of the NF‐κB transcription factor. Concurrently, the CD36 receptor plays a pivotal role in facilitating the internalisation of oxLDL into macrophages, a key step in foam cell formation [35]. Based on in silico studies from Li et al., utilising docking and fluorescence immunocytochemistry elucidates that the ω‐carboxyl group on oxLDL is crucial for its interaction with LOX‐1, subsequently initiating intracellular Src‐JNK/ERK1/2 signal transduction pathways [36]. TLR4 stimulation enhances LOX‐1 receptor activity via the Erk1/2 signalling pathway. This mechanism subsequently leads to increased uptake of oxLDL by macrophages [37].

The initiation of this inflammatory signalling cascade is triggered by the elevated production of reactive oxygen species (ROS) upon the binding of oxLDL to CD36 and LOX‐1. This process is mediated by NADPH oxidase activation, generating superoxide anions (O_2_^−^). Furthermore, hydrogen peroxide (H_2_O_2_) modulates NF‐κB activation by promoting IκB kinase (IKK) activation, which subsequently drives the phosphorylation and degradation of IκBα [38–44]. The binding of oxLDL to LOX‐1, CD36 and SR‐A activates several intracellular signalling pathways, such as triggering the phosphorylation of MAPKs, specifically p38 MAPK, ERK 1/2 and JNK 1/2 as well as NF‐κB inducing kinase (NIK) and IKK. These pathways regulate pleiotropic transcription factors that control the production of NO and proinflammatory cytokines [45–50]. Signalling via ERK 1/2 [51–53], MAPK and JNK 1/2 leads to the activation of NF‐κB cells [54–57]. Moreover, the interaction between oxLDL and CD36 initiates a signalling cascade that involves Src‐family kinases and Vav family guanine nucleotide exchange factors [58].

### 2.5. Activation of NF‐κB and Production of Inflammatory Cytokines

MAPK activation leads to the phosphorylation and degradation of IκB‐α, an inhibitor of NF‐κB, thereby allowing NF‐κB to translocate into the nucleus and initiate the transcription of inflammatory genes [50, 59]. In this state, activated JNK induces c‐Jun phosphorylation, which synergises with NF‐κB to upregulate the transcription of inflammatory genes [60, 61]. Concurrently, the activation of ERK is mediated by tumour progression locus 2 (TPL‐2), a MAP 3‐kinase. This kinase is regulated by IKK, resulting in the degradation of NF‐κB1 p105 and the subsequent activation of NF‐κB [62–64]. Inside the nucleus, NF‐κB forms dimers and binds to specific loci on the DNA called κB sites. These sites are situated within the regulatory regions of genes. The binding of NF‐κB triggers transcription, where it helps recruit other proteins, such as coactivators, to drive the transcription of proinflammatory genes [65]. NF‐κB functions as a central regulator in atherosclerosis by controlling the transcription of adhesion molecules, chemokines and proinflammatory cytokines that sustain endothelial activation, leukocyte recruitment, chronic vascular inflammation and progressive plaque development [66].

The activation of TLRs on macrophages, specifically TLR2 and TLR4, by oxLDL binding triggers proinflammatory signalling pathways via the activation of NF‐κB [67]. The interaction between oxLDL and TLR2 or TLR4 can also engage coreceptors, including CD36 [2]. Upon binding to oxLDL, TLR2 and TLR4 undergo dimerisation and recruit adaptor proteins, specifically myeloid differentiation primary response 88 (MyD88) and TIR‐domain–containing adaptor‐inducing interferon‐β (TRIF) [67–69]. This process leads to the activation of downstream signalling molecules, namely interleukin‐1 receptor‐associated kinase (IRAK) and TNF receptor–associated factor 6 (TRAF6) [70, 71]. Subsequently, this triggers kinase signalling cascades, specifically MAPKs such as ERK, JNK and p38, which in turn activate the NF‐κB pathway [69, 71, 72].

### 2.6. Activation of the NLRP3 Inflammasome and Interleukin Production

The transcriptional activity of NF‐κB promotes the expression and secretion of various proinflammatory cytokines, such as TNF‐α and IL‐6 [73]. Additionally, NF‐κB mediates the regulation of IL‐18 and IL‐1β via the NLRP3 inflammasome. During the priming step of the NLRP3 inflammasome, NF‐κB drives the transcriptional upregulation of NLRP3, pro‐IL‐1β and pro‐IL‐18 [74]. This is followed by the activation step, which involves various cellular stress signals, including the generation of ROS, potassium efflux and lysosomal disruption. Mitochondrial dysfunction and xanthine oxidase activity further contribute to ROS generation [75]. Lysosomal rupture and the subsequent release of cathepsin B contribute to the activation of the NLRP3 inflammasome [75]. The assembly of the NLRP3 inflammasome complex—comprising NLRP3, ASC and pro‐caspase‐1 (pro‐CASP1) leads to the activation of CASP1 [75]. Activated CASP1 mediates the cleavage of pro‐IL‐1β and pro‐IL‐18 into their mature forms (IL‐1β and IL‐18), which are subsequently secreted by the macrophages [76].

### 2.7. Chronic Effects and Foam Cell Development

**FIGURE 2:**
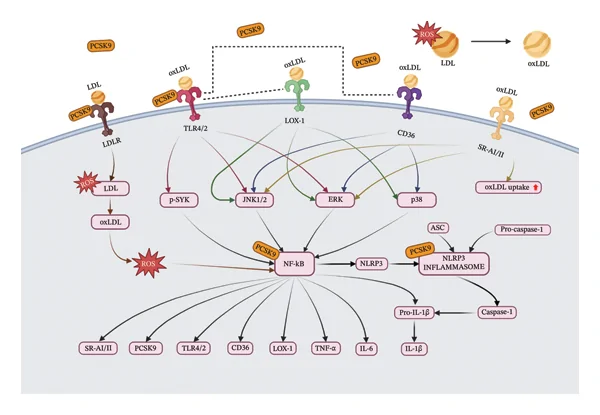
Mechanisms of oxLDL‐induced inflammation and foam cell formation in atherosclerosis. oxLDL is internalised by scavenger receptors (CD36, LOX‐1, SR‐A) on macrophages, promoting foam cell formation. oxLDL binding also activates receptors (TLR4/2, LOX‐1), triggering kinase signalling (SYK, JNK, ERK, p38) and NF‐κB activation. This leads to proinflammatory cytokine (TNF‐α, IL‐6, pro‐IL‐1β) transcription. Concurrently, NLRP3 inflammasome assembly activates caspase‐1, which cleaves pro‐IL‐1β into active IL‐1β. The pathway also shows a link to PCSK9‐mediated LDLR regulation. Created in BioRender. Hestu, M. (2026) https://BioRender.com/bv7hkys.

Additionally, the interaction between oxLDL and CD36 induces the production of monocyte chemoattractant protein‐1 (MCP‐1) [77]. Binding of oxLDL to CD36 promotes MCP‐1 expression primarily through ROS‐dependent NF‐κB activation [43, 78], while JNK‐mediated signalling further amplifies transcriptional activity associated with monocyte recruitment and inflammatory progression [79]. This chemokine plays a critical role in the early stages of atherosclerosis by facilitating monocyte migration and infiltration into the intima, where they differentiate into macrophages [80]. Consequently, macrophages exposed to high levels of oxLDL exhibit upregulated expression of proinflammatory cytokines [81], including TNF‐α, IL‐6 and IL‐1β. Notably, TNF‐α and IL‐1β promote foam cell formation by inhibiting lipid catabolism, leading to the intracellular retention of cholesterol and triglycerides. Thus, under conditions of chronic oxLDL exposure, the macrophage proinflammatory state is sustained [82]. oxLDL uptake via SRs activates the inflammasome and proinflammatory signalling as shown in Figure 2.

## 3. PCSK9 Contribution in Foam Cell Formation Process

### 3.1. Cellular Source of PCSK9 in the Vasculature

PCSK9 acts as a proinflammatory accelerator in macrophages [83]. Macrophages and endothelial cells do not initiate endogenous PCSK9 production. Instead, they rely on exogenous PCSK9 exposure. A comparative analysis by Ferri et al., utilising qPCR and Western blotting, revealed striking differences in expression profiles among vascular cell types. Human smooth muscle cells (hSMCs) exhibited high levels of PCSK9 mRNA and protein expression, comparable to HepG2 cells (positive control). In contrast, PCSK9 expression was undetectable in endothelial cells (HUVECs), monocytes and macrophages. These findings confirm that macrophages and endothelial cells do not produce PCSK9 endogenously but are instead targets of exogenous PCSK9. Further validation in human carotid atherosclerotic plaques confirmed the presence of this protein in situ. Specifically, immunohistochemical staining demonstrated that PCSK9 distribution colocalises with α‐SMA–positive areas (a smooth muscle marker) but is distinct from CD68 localisation (a macrophage marker). This establishes hSMCs as the primary reservoir of PCSK9 within atherosclerotic lesions. Consequently, PCSK9 secreted by hSMCs operates via a paracrine mechanism to degrade LDLR on the surface of neighbouring monocytes and macrophages. This is evidenced by a drastic reduction in LDLR expression, which significantly impairs the LDL uptake capacity of these immune cells [7].

### 3.2. PCSK9 Inflammatory Signalling via TLR4/NF‐κB

Jaén et al. described a mechanism in which extracellular PCSK9 acts as a ligand for the TLR4 receptor, thereby initiating the NF‐κB signalling cascade. Their analysis revealed that PCSK9 induction in macrophages provokes a rapid surge in IκB‐α phosphorylation within the first 5–15 min. This phosphorylation triggers the degradation of IκB‐α, occurring concurrently with increased p65 phosphorylation, which signifies the full activation of the canonical NF‐κB pathway. Consequently, this transcriptional activation leads to the upregulation of key proinflammatory genes, specifically NLRP3, IL‐1β and TNF‐α. Furthermore, PCSK9 exposure was shown to activate the NLRP3 inflammasome, promoting the processing of pro‐CASP1 into active CASP1, evidenced by a significant increase in the p10 subunit and inducing oxidative stress. This accumulation of proinflammatory signals ultimately results in apoptosis. This was molecularly validated by the emergence of cleaved CASP3, the apoptotic executioner, starting at 60 min and intensifying by the second hour. The pivotal role of TLR4 as the primary gatekeeper in this process was confirmed using the selective inhibitor TAK242. This inhibition effectively abolished the entire signalling cascade from the initial phosphorylation of NF‐κB and p38 MAPK to CASP3 activation, demonstrating that PCSK9‐induced inflammation and macrophage cell death are strictly dependent on TLR4 mediation [84].

Research conducted by Tang et al. has shown that PCSK9 operates by triggering the TLR4 and NF‐κB signalling pathways; higher levels of PCSK9 were observed to boost TLR4 expression, resulting in a greater concentration of these receptors on the cell surface and intensifying the inflammatory reaction. Specifically, an excess of PCSK9 prompted a notable increase in IκB phosphorylation (p‐IκB) and the breakdown of total IκB. The reduction of this inhibitory protein was directly associated with the augmented nuclear retention of the p65 subunit, which is indicative of the complete activation of proinflammatory gene transcription [83]. The heightened TLR4 activity induced by exposure to PCSK9 also encourages a significant surge in the absorption of oxLDL by macrophages [37]. Furthermore, it has been reported that the accumulation of internalised oxLDL itself directly stimulates cellular mechanisms within macrophages to enhance the creation of PCSK9, affecting both the transcriptional and translational processes [85].

**FIGURE 3:**
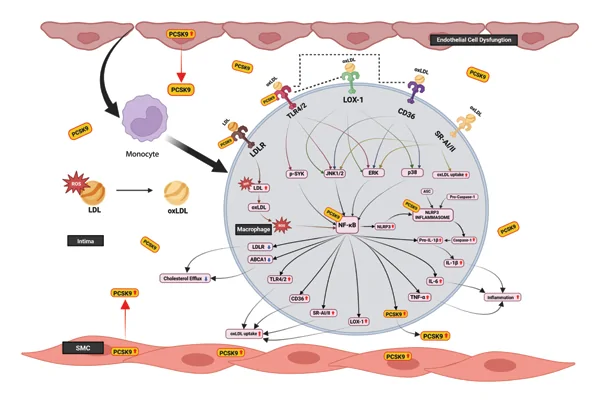
Proprotein convertase subtilisin/kexin type 9 (PCSK9), which is primarily released by vascular smooth muscle cells (SMCs), exerts paracrine effects on infiltrating monocytes and macrophages within the arterial intima. Through interaction with Toll‐like receptor 4/2 (TLR4/2), PCSK9 amplifies macrophage inflammatory activity by triggering downstream signalling cascades involving SYK, JNK1/2, ERK and p38 MAPK, ultimately converging on nuclear factor‐κB (NF‐κB). Activation of NF‐κB drives the expression of proinflammatory mediators, including TNF‐α, IL‐6 and pro‐IL‐1β, enhances the expression of scavenger receptors such as LOX‐1, CD36 and SR‐A I/II and facilitates assembly of the NLRP3 inflammasome. Subsequent activation of caspase‐1 enables the processing and secretion of mature IL‐1β, thereby intensifying vascular inflammatory responses. In parallel, PCSK9 promotes oxLDL uptake while impairing cholesterol efflux by suppressing ATP‐binding cassette transporter A1 (ABCA1) and accelerating low‐density lipoprotein receptor (LDLR) degradation. Collectively, these interconnected mechanisms favour lipid accumulation, persistent inflammatory signalling and macrophage differentiation into foam cells, contributing to the progression of atherosclerotic plaques. Created in BioRender. Hestu, M. (2026) https://BioRender.com/qvthcpr.

PCSK9‐induced inflammation exacerbates the production of proinflammatory cytokines, including TNF‐α, IL‐1 and IL‐6. In a study using THP‐1 macrophages, Ricci et al. demonstrated that PCSK9 mediates this inflammatory response and upregulates TNF‐α via the JAK/STAT and SREBP signalling axes. The involvement of these mechanisms was confirmed through inhibition assays; blocking the JAK/STAT pathway with a JAK inhibitor, or the SREBP pathway with fatostatin, significantly attenuated TNF‐α levels, returning them to baseline. Moreover, exposure to hPCSK9 (2.5 μg/mL) triggered a massive 31.1‐fold increase in TNF‐α mRNA expression in wild‐type (LDLR+/+) macrophages, whereas LDLR‐deficient (LDLR−/−) macrophages showed only a marginal increase. While several studies demonstrate that activated macrophages can produce PCSK9 under inflammatory conditions, other reports suggest that hepatic‐derived PCSK9 remains the predominant contributor to circulating PCSK9 levels, indicating that macrophage‐derived PCSK9 may exert predominantly local rather than systemic effects [86, 87]. Nevertheless, accumulating evidence indicates that PCSK9 plays a substantial role in macrophage‐mediated inflammatory responses within atherosclerotic lesions. PCSK9 amplifies inflammatory signalling in macrophages by promoting SR expression, enhancing oxLDL uptake, activating NF‐κB‐dependent cytokine production and suppressing cholesterol efflux pathways, thereby accelerating foam cell formation [88, 89]. These findings indicate that the interaction between PCSK9 and LDLR is crucial for amplifying the inflammatory response, highlighting LDLR as a primary target of PCSK9 [5]. Although numerous studies have demonstrated the proinflammatory role of PCSK9 in macrophage activation and foam cell formation, several inconsistencies remain regarding the extent of PCSK9 expression across different macrophage phenotypes and experimental settings. Some investigations report substantial upregulation of PCSK9 under inflammatory stimulation, whereas others observe comparatively modest expression changes, suggesting that PCSK9‐mediated inflammatory responses may be highly context‐dependent. These discrepancies may arise from variations in cellular models, oxLDL concentrations, inflammatory stimuli, exposure duration and methodological approaches employed across studies. Collectively, these findings indicate that the contribution of PCSK9 to macrophage inflammatory signalling is likely influenced by both microenvironmental and experimental conditions, thereby warranting further mechanistic clarification [5, 90, 91]. Mechanistic overview of PCSK9‐mediated inflammatory signalling pathways contributing to macrophage foam cell formation during atherosclerosis progression, as seen in Figure 3.

### 3.3. Amplification of oxLDL Uptake and SR Expression

A study by Ding et al. demonstrated that inducing an inflammatory state in macrophages using TNF‐α and PCSK9 stimulates the production of PCSK9 as well as the SRs LOX‐1, SR‐A and CD36. The data revealed a dose‐dependent increase in protein expression, with the most pronounced elevation observed in LOX‐1. Consequently, this led to a fivefold increase in the rate of oxLDL internalisation, a process predominantly mediated by LOX‐1 compared to other receptors. Conversely, the same treatment resulted in the downregulation of LDLR expression. Furthermore, PCSK9 synthesis under these conditions was shown to be mediated by ROS through NADPH oxidase activity [26].

### 3.4. Impairment of Cholesterol Efflux via ABCA1 Degradation

Under physiological conditions, ABCA1 plays a pivotal role in facilitating cholesterol efflux from macrophages to apolipoprotein A‐I (ApoA‐I) [92]. However, exposure to PCSK9 disrupts this homeostasis by significantly downregulating ABCA1 expression. Adorni et al. demonstrated that this negative regulation is LDLR‐dependent, indicating that PCSK9 does not act independently but requires the LDLR as an obligate mediator to form a complex that triggers ABCA1 degradation. Consequently, the loss of ABCA1 function impairs cholesterol efflux, leading to the intracellular retention of lipids internalised via oxLDL. Ultimately, this uncontrolled lipid accumulation accelerates the transformation of macrophages into foam cells [9]. Furthermore, the reduction in LDLR mRNA levels following PCSK9 exposure suggests that PCSK9 limits LDLR availability not only through lysosomal degradation but also via suppression at the transcriptional level. This inhibitory mechanism involves decreased expression of *SREBF1*, which correlates directly with the downregulation of its downstream target genes, *FASN* and *HMGCR*, both of which are crucial for fatty acid and cholesterol synthesis. Consequently, macrophages exhibit an impaired capacity for de novo lipid synthesis, thereby exacerbating their vulnerability to lipid stress [84].

A specific surge in mitochondrial ROS production is initiated by exposure to oxLDL, which induces mitochondrial dysfunction. While oxLDL promotes the uptake and mitochondrial import of long‐chain fatty acids, it simultaneously inhibits ATP5A. This metabolic imbalance leads to fatty acid accumulation and the repurposing of the electron transport chain to generate excessive amounts of superoxide. Consequently, this oxidative stress acts as an upstream signal that activates the NF‐κB transcription factor, triggering a cascade of proinflammatory mediator synthesis [78]. The ligation of PCSK9 to the TLR4 receptor has been shown to induce ROS production in a dose‐dependent manner, where increased concentrations correlate directly with a surge in ROS levels. Furthermore, TLR4 acts as a crucial mediator in the interaction between PCSK9 and LDLR. This synergistic interplay significantly amplifies the inflammatory response and exacerbates the pathological state of macrophages. Consequently, the elevated intracellular ROS levels drive the rate of cellular apoptosis [84]. PCSK9 accelerates foam cell development by coordinating TLR4/NF‐κB activation with increased SR expression and reduced cholesterol efflux.

## 4. Bioactive Compounds Target the Inhibition of the M1 Differentiation Stage

### 4.1. Salvianolic Acid B (Sal B)

Sal B, a major polyphenolic part of *Salvia miltiorrhiza* (Danshen), displays significant antiatherogenic properties by targeting macrophage polarisation and function [92]. In vitro investigations using RAW264.7 macrophages indicate that Sal B effectively suppresses M1 polarisation while promoting features associated with the anti‐inflammatory M2 phenotype [14]. The mechanisms underlying these effects involve several key pathways:

a. Inhibition of NF‐κB signalling: Sal B exerts a potent anti‐inflammatory effect by blocking the nuclear translocation of the NF‐κB p65 subunit. This inhibition represses the transcription of proinflammatory genes, leading to a marked downregulation of classic M1 markers, including iNOS, TNF‐α and interleukin‐6 (IL‐6). In LPS‐stimulated RAW264.7 macrophages and primary peritoneal macrophages, Sal B significantly inhibits the phosphorylation and nuclear translocation of the NF‐κB p65 subunit. This leads to the downregulation of proinflammatory M1 markers, including iNOS, TNF‐α and IL‐6 [14, 93]. The other studies in ApoE‐deficient (ApoE−/−) mice fed a high‐fat diet, Sal B treatment reduced atherosclerotic plaque area and stability, correlating with decreased NF‐κB activation and lower expression of TNF‐α and IL‐6 within plaques [94], and Sal B enhances autophagic flux, a process modulated via its interaction with the NF‐κB pathway. This increased autophagy helps clear damaged cellular components, thereby alleviating cellular stress and contributing to the resolution of inflammation. Experiments with oxLDL–exposed RAW264.7 macrophages, a well‐established cellular model of atherosclerosis, show that Sal B effectively stimulates autophagy. Indicators of this activity include the elevated levels of the autophagy markers LC3‐II and Beclin‐1, coupled with a reduction in p62, a protein that accumulates when autophagy is disrupted. Research indicates this process is linked to Sal B’s ability to suppress the NF‐κB signalling pathway. The functional importance of autophagy was confirmed when blocking it with specific inhibitors or genetic tools markedly reduced Sal B’s anti‐inflammatory benefits. This evidence establishes that Sal B significantly reduces inflammation primarily through activating autophagy, which is a core mechanism rather than a side effect [14, 95]. This pathway’s importance is confirmed in living organisms. In a mouse model of myocardial ischemia/reperfusion (mimicking a heart attack), Sal B administration provided measurable protection to heart tissue, limiting injury and maintaining function. Examination of the heart muscle showed that these positive results were linked to increased autophagy activity and lower inflammation markers. These in vivo findings support previous cell studies, affirming that Sal B’s enhancement of autophagy is a crucial and essential part of its heart‐protective effect after injury [96].
b. Promotion of M2 Polarisation: Concurrently with M1 suppression, Sal B actively promotes the alternative, anti‐inflammatory M2 polarisation. This is evidenced by the upregulation of M2‐associated markers such as arginase‐1 (Arg‐1) and interleukin‐10 (IL‐10), indicating a shift towards tissue repair and resolution functions. Sal B directly promotes the anti‐inflammatory, tissue‐repair functions of macrophages. In laboratory studies using RAW264.7 cells, when treated with IL‐4 (a signal that starts the M2 switch), Sal B works cooperatively to significantly amplify the production of characteristic M2 proteins like arginase‐1 (Arg‐1) and the anti‐inflammatory cytokine interleukin‐10 (IL‐10). Even more compelling is its action in inflamed settings: When macrophages are activated by LPS into a proinflammatory M1 state, Sal B can actively reprogram them. It shifts their functional profile from a damaging, M1‐dominated state back towards a beneficial, M2‐like phenotype, demonstrating a capacity to reverse inflammation‐driven polarisation [14]. Studies on rats suffering from diabetic atherosclerosis, a condition marked by significant inflammation, showed that treatment with Sal B resulted in observable changes within the compromised blood vessels. Primarily, it increased the count of macrophages expressing CD206, a known M2 subtype indicator, inside the atherosclerotic plaques. This cellular shift led to direct therapeutic advantages: The plaques became more stable (reducing the chance of rupture and clot formation), and systemic inflammatory markers decreased throughout the body [86]. These findings confirm that Sal B’s ability to promote M2 macrophages, observed in cellular research, provides tangible benefits in a living organism, helping to stabilise local plaques and manage overall inflammation [95].
c. Modulation of the Akt/mTOR Pathway: Sal B also inhibits the Akt/mTOR signalling axis, which is a central regulator of cellular metabolism and inflammatory responses. This inhibition further reinforces its overall anti‐inflammatory and polarisation‐shifting effects. Sal B targets a fundamental cellular signalling pathway known to regulate inflammation and metabolism: the Akt/mTOR cascade [97]. Laboratory studies using LPS‐stimulated macrophages demonstrate that Sal B effectively reduces the activation (phosphorylation) of both Akt and mTOR proteins. This specific inhibitory action is a key mechanism behind its observed cellular effects. By putting a brake on this pathway, Sal B achieves two important outcomes: It helps suppress the expression of proinflammatory M1 markers and concurrently stimulates the cellular recycling process of autophagy [14, 98]. The significance of targeting this pathway is strongly supported by evidence from animal models of disease. Research on the importance of targeting this pathway is robustly supported by evidence from animal disease models. Research involving mice with cerebral ischemia (stroke) demonstrated that Sal B’s neuroprotective capacity, which resulted in reduced brain damage, was mechanistically linked to its impact on this pathway [99].

### 4.2. Curcumin

Curcumin, the primary bioactive compound in turmeric (*Curcuma longa*), demonstrates broad antiatherogenic activity by regulating macrophage polarisation and function. This effect is well‐established across both in vivo and in vitro models. Research has shown that curcumin effectively shifts macrophages away from a proinflammatory M1 state towards a more anti‐inflammatory profile, a key mechanism in mitigating vascular inflammation and preventing atherosclerotic plaque development.

Yan et al. demonstrated through in vivo studies that curcumin effectively suppresses M1 macrophage polarisation. This was evidenced by the downregulation of M1 markers (iNOS and CD86) and proinflammatory cytokines (TNF‐α, IL‐1β and IL‐6), coupled with an upregulation of the anti‐inflammatory cytokine IL‐10. Mechanistically, this polarisation process is regulated by AMPK signalling. In rat hearts, curcumin was observed to attenuate the elevated expression of the p‐AMPK/AMPK ratio. Meanwhile, in vitro experiments on mouse bone marrow macrophages (BMMs) revealed that curcumin directly promotes AMPK phosphorylation (p‐AMPK). This activation significantly inhibits M1 polarisation, a critical step in mitigating inflammation and preventing foam cell formation [13].

Beyond modulating polarisation, curcumin exhibits several complementary antiatherogenic actions on macrophages. It regulates SR activity, notably reducing oxLDL‐induced expression of CD36 by suppressing the phosphorylation of p38 MAPK and JNK [100, 101]. Curcumin also broadly suppresses the production of proinflammatory cytokines in M1 macrophages [102] and helps restore cholesterol transport homeostasis, likely through the modulation of ABC transporters and lipid metabolic pathways.

In summary, curcumin exerts its antiatherogenic influence through a multifaceted approach: It directly activates AMPK to repress proinflammatory M1 polarisation, concurrently dampens cytokine production, limits modified lipid uptake via SR regulation and promotes cholesterol efflux, collectively contributing to reduced macrophage‐driven inflammation and foam cell formation.

### 4.3. Quercetin

Quercetin, a flavonoid abundant in many fruits, vegetables and herbal remedies, displays important antiatherogenic qualities through its influence on key processes in macrophage lipid metabolism, inflammation and cellular ageing. These actions are supported by evidence from both laboratory and animal studies. Research in cellular models, particularly using RAW264.7 macrophages, shows that quercetin helps prevent damage caused by oxidised LDL. It improves cell survival, lessens the build‐up of fats (evident from reduced Oil Red O and Filipin staining) and decreases overall cholesterol. At a molecular level, quercetin boosts the production of cholesterol removal proteins ABCA1 and ABCG1, along with their controlling factor LXR‐α. At the same time, it reduces levels of the lipid‐related protein PCSK9 and proteins linked to ageing—p53, p21 and p16. This combined effect supports the removal of cholesterol from cells, hinders the development of foam cells and slows down cellular senescence. Quercetin also raises the activity of the antioxidant enzyme superoxide dismutase (SOD) and lowers malondialdehyde (MDA), pointing to a strengthening of the cell’s antioxidant defences [103].

Quercetin has been shown to increase ABCA1 in THP‐1 macrophages and in apoE‐deficient mice by working through the LXR‐α signalling route, thereby encouraging cholesterol removal from macrophages [104, 105]. It also lowers PCSK9 expression in liver cells, aiding better lipid control [106–108]. Further research indicates that quercetin can dampen inflammation by affecting the TLR4/NF‐κB pathway, adding to its potential in countering atherosclerosis. In short, quercetin acts against atherosclerosis via several connected mechanisms: It increases ABCA1/ABCG1 and LXR‐α to help clear cholesterol, lowers PCSK9 to assist lipid regulation, diminishes oxidative stress and markers of ageing and adjusts inflammatory signalling. Together, these actions help stop macrophages turning into foam cells and slow the advance of atherosclerosis. Table 1 shows the effects of Sal B, curcumin and quercetin activity to inhibition macrophage polarisation.

**TABLE 1:** Effects of salvianolic acid B, curcumin and quercetin on macrophage polarisation, inflammation and associated molecular pathways.

Compounds	Biological activity	Methods
Salvianolic acid B (Sal B)	1 Inhibition of NF‐κB signalling and suppression of M1 polarisation:	In vitro: LPS‐stimulated RAW264.7 macrophages [14]
	• Blocks NF‐κB p65 subunit nuclear translocation.	In vivo: male mice LDLR −/− [93]
	• Downregulates proinflammatory M1 markers (iNOS, TNF‐α, IL‐6).	
	• Decreases atherosclerotic plaque area and improves stability	
	2 Induction of autophagy	In vitro: LPS‐stimulated RAW264.7 macrophages [14]; JS1, the mouse immortalised stellate cell lines and LX2 human hepatic stellate cell lines [95]
	• Enhances autophagic flux (increases LC3‐II, Beclin‐1; decreases p62).	In vivo: mice with myocardial ischemia/reperfusion injury [96]
	• Anti‐inflammatory effect is autophagy‐dependent	
	• Provides cardioprotective effects associated with increased autophagy and reduced inflammation	
	3 Promotion of M2 polarisation	In vitro: LPS‐stimulated RAW264.7 macrophages [14]
	• Synergises with IL‐4 to upregulate M2 markers (Arg‐1, IL‐10).	
	• Reprograms LPS‐stimulated macrophages from M1 to an M2‐like phenotype.	
	• Increases CD206+ M2 macrophages within plaques.	
	• Promotes plaque stability and reduces systemic inflammation	
	4 Modulation of the Akt/mTOR pathway	In vitro: RAW264.7 cells LPS‐stimulated macrophages [98]
	• Inhibits phosphorylation of Akt and mTOR.	In vivo: Mice with cerebral ischemia (stroke) [99]
	• Contributes to M1 suppression and autophagy induction	
	• Neuroprotection linked to PI3K/Akt/mTOR inhibition and modulation of microglial	

Curcumin	• Suppresses M1 macrophage polarisation by downregulating M1 markers (iNOS, CD86) and proinflammatory cytokines.	In vitro: bone marrow macrophages; in vivo: wild‐type (C57BL/6) mice using bone marrow macrophages [13]
	• Attenuates the elevated expression of the p‐AMPK/AMPK ratio	
	• Regulates scavenger receptor activity, reducing oxLDL‐induced expression of CD36	

Quercetin	• Reduces oxLDL‐induced lipid	In vitro *cell* model: RAW264.7 murine macrophages
	• Enhances cholesterol efflux via the upregulation of ABCA1, ABCG1 and LXR‐α	Total cholesterol (TC) assay; SOD and MDA biochemical assays [103]
	• Antioxidant effect (↑ SOD, ↓ MDA)	
	• Improves macrophage viability	

## 5. Controlling Foam Cells With Natural Products

### 5.1. Curcumin

Curcumin, the active component in turmeric, combats foam cell formation through multiple pathways. In laboratory studies using immune cells, concentrations between 5 and 80 μM have been shown to inhibit the expression of the cholesterol uptake receptor CD36 and reduce proinflammatory cytokines like TNF‐α and IL‐6 [109].

These findings are supported in animal models. In mice prone to atherosclerosis, a diet supplemented with 40–80 mg/kg BW curcumin significantly decreases lipocalin‐2 (LCN2) and inflammatory markers within lesions [110]. In human clinical studies, curcumin has demonstrated tangible benefits. Daily supplementation with 70 mg to 1500 mg of curcuminoids has been shown to lower systemic inflammation (reducing TNF‐α and IL‐6) and improve vascular function in individuals with metabolic syndrome [111]. While direct imaging of foam cells in humans remains limited, these biochemical and functional improvements strongly support its therapeutic potential.

### 5.2. Fisetin

Found in strawberries and other fruits, fisetin targets the NLRP3 inflammasome—an inflammatory complex linked to plaque progression. In vitro, doses of 1–70 μM inhibit inflammasome assembly and reduce the release of inflammatory cytokines such as IL‐1β and IL‐18 in macrophage models [112]. In animal studies, oral administration of 20 mg/kg/day in ApoE^−/−^ mice attenuates disease progression, reduces necrotic areas within plaques, lowers oxidative stress and inhibits PCSK9 [113]. Although clinical research on fisetin for cardiovascular disease is still emerging, early phase trials in other conditions have used doses up to 1400 mg/day in humans, demonstrating safety and measurable bioavailability—laying a foundation for future heart‐related studies [114].

### 5.3. Myricetin

This flavonoid, present in berries and tea, primarily limits foam cell formation by blocking oxidised cholesterol uptake. In cellular experiments, concentrations of 5–50 μM downregulate CD36 receptor expression and reduce lipid accumulation in macrophages [115]. In vivo studies showed 100 mg/kg mice myricetin caused a decrease in inflammatory cytokines and at doses 200 mg/kg protective effects via enhancing Nrf2/HO‐1 and inhibiting I*κ*B*a*/NF*κ*B [116, 117]. While no direct clinical trials have yet evaluated myricetin’s effect on atherosclerosis, population studies associate higher intake of dietary flavonols—which include myricetin—with reduced cardiovascular mortality, suggesting a protective role worthy of further targeted investigation [118].

### 5.4. Zerumbone

Zerumbone is a bioactive compound found in the *Zingiber zerumbet* Smith plant. According to research conducted by Eguchi et al., in THP‐1 macrophage cells induced with 12‐O‐tetradecanoylphorbol‐13‐acetate (TPA), it significantly increased the expression of LOX‐1 mRNA compared to the control group, thereby triggering inflammatory processes. In a dose‐dependent manner, increasing concentrations of zerumbone—1, 5, 10 and up to 20 μM—correlated linearly with a decrease in LOX‐1 receptor mRNA levels. The effectiveness of this compound was determined to have an IC50 at a concentration of 9.4 μM. Zerumbone also demonstrated a strong ability to completely block the transcription of SR‐A and SR‐PSOX mRNA. Meanwhile, the expression of the CD36 receptor showed a significant decrease of 85%. At a concentration of 10 μM, zerumbone was found to reduce the activity of activator protein‐1 (AP‐1) and NF‐κB, with the most dominant suppression observed in AP‐1 [119]. Table 2 shows a side‐by‐side view of natural antiatherosclerotic agents.

**TABLE 2:** Experimental evidence for curcumin, fisetin, myricetin and zerumbone in targeting atherosclerosis.

Compounds	Biological activity	Methods
Curcumin	• Inhibits the expression of cholesterol	In vitro: Human monocytic cell line (THP‐1) and foam cell [109]
	• Reduces atherosclerosis via decreases LCN2	In vivo: wild‐type C57BL/6J and ApoE^−/−^ mice [110]
	• Decreases macrophage infiltration and inflammatory markers	Clinical trial: individuals with metabolic syndrome [111]
	• Lowers systemic inflammation	

Fisetin	• Inhibits oxidative stress and PCSK9	In vivo: wild‐type C57BL/6J and ApoE^−/−^ mice male ApoE^−/−^ mice [113].
	• Attenuates atherosclerosis progression	

Myricetin	• Downregulates CD36 receptor expression	In vitro: macrophage models [115]
		In vivo: male C57BL/6 J mice [116]; mice with STZ induction [117]

Zerumbone	• Suppressed TPA‐induced mRNA expression of multiple scavenger receptors (LOX‐1, SR‐A, SR‐PSOX, CD36)	In vitro: THP‐1 human monocytic cells [119]
	• Blocked cellular uptake of Dil‐acLDL	
	• Inhibited transcriptional activities of AP‐1 and NF‐κB	

## 6. Bioactive Compounds as Inhibitors of PCSK9 Expression

### 6.1. Berberine and Resveratrol

The isoquinoline alkaloid berberine has emerged as a particularly strong candidate. It works through multiple channels: It activates the cellular energy sensor AMPK, which in turn suppresses the cholesterol‐regulating protein SREBP2—a primary driver of PCSK9 gene expression [120, 121]. Furthermore, it helps recycle LDLRs on the liver’s surface, making them less vulnerable to degradation by PCSK9.

In vitro studies using HepG2 cells, berberine at concentrations between 40 μM, effectively lowered PCSK9 production. This translated to animals, where mice fed a high‐fat diet and given berberine (200 mg/kg daily) showed reduced blood levels of both PCSK9 and LDL cholesterol [122].

Similarly, resveratrol, the famed compound in red grapes, inhibits the same SREBP2 pathway but via a different mechanism, stabilising a protein complex that keeps SREBP2 inactive. While cell (10 and 20 μM) [123] and animal models (using doses around 40 mg/kg/day in rats) consistently show PCSK9 reduction [124]. The human data remain preliminary. High‐dose resveratrol supplements (1000–2000 mg/day) have shown modest lipid improvements [125, 126], but research specifically confirming its direct PCSK9‐lowering effect in people is still needed.

### 6.2. Flavonoids: Quercetin and EGCG

Found in foods like apples and onions, quercetin takes a different tactical approach. It primarily inhibits a transcription factor called HNF1α, another key switch for the PCSK9 gene [107, 108]. Animal models, such as ApoE‐deficient mice given 75 mg/kg/day, support its PCSK9‐reducing ability [106]. In humans, while standard supplemental doses (500–1000 mg/day) are linked to better cardiovascular markers [127], we await trials designed to measure PCSK9 directly as the main outcome.

EGCG, the major antioxidant in green tea, may work further down the line, potentially interfering with the enzymatic maturation of the PCSK9 protein itself [128]. Cell studies and research in hamsters support this activity [129]. The consistent LDL‐lowering effect of green tea consumption seen in meta‐analyses hints at a relevant mechanism, although clinical trials rarely measure PCSK9 levels specifically.

### 6.3. Curcumin

The active component of turmeric, curcumin, influences PCSK9 through anti‐inflammatory action, suppressing the NF‐κB signalling pathway that can upregulate its expression [130]. It may also tweak microRNAs that regulate PCSK9. Studies in cells and hyperlipidaemic rats (starting from 100 mg/kg/day) are positive [131]. While the preclinical science is robust, translating this to widespread clinical practice faces real‐world hurdles. A significant advantage is their multitargeted approach and generally favourable safety profile, which allows for potential synergy with drugs like statins. However, major challenges persist. The most pressing issue is bioavailability; compounds like resveratrol and curcumin are poorly absorbed on their own, necessitating advanced delivery systems. There is also a translational gap between effective concentrations in cell studies and what is achievable in human blood. Perhaps the most critical bottleneck is the lack of large‐scale, long‐term human trials where PCSK9 reduction is the primary goal, rather than a secondary observation. There is also a translational gap between effective concentrations in cell studies and what is achievable in human blood. Perhaps the most critical bottleneck is the lack of large‐scale, long‐term human trials where PCSK9 reduction is the primary goal, rather than a secondary observation of the effects of administered PCSK9 inhibitors.

### 6.4. Brazilin

Brazilin, a natural compound extracted from the heartwood of *Caesalpinia sappan* L., has been identified as a novel small‐molecule inhibitor of PCSK9. Its mechanism involves directly disrupting the interaction between PCSK9 and the low‐density lipoprotein receptor (LDLR), as evidenced by integrated in silico and in vitro analyses. Using pharmacophore‐based virtual screening of the Indonesian HerbalDB database, brazilin was selected as a promising candidate due to its predicted high binding affinity for PCSK9. Molecular docking simulations performed with Pyrx 0.98 confirmed this, showing that brazilin had the strongest binding affinity (−9.0 kcal/mol) among the screened compounds. The interaction is characterised by several hydrogen bonds and hydrophobic contacts within the PCSK9 active site, particularly in the region essential for LDLR engagement.

In vitro validation with a competitive PCSK9–LDLR binding assay demonstrated that brazilin inhibits this protein–protein interaction in a dose‐dependent manner, yielding an IC_50_ value of 2.19 μM. The compound showed greater potency than the crude *C. sappan* extract, confirming its role as the primary bioactive constituent [132]. Unlike many natural compounds that lower PCSK9 activity by suppressing its expression, brazilin acts through direct, competitive inhibition at the protein level. This distinct mechanism offers a complementary approach to existing lipid‐lowering strategies and positions brazilin as a promising lead molecule for the development of orally administered PCSK9 inhibitors. Table 3 showed PCSK9‐targeting natural compounds.

**TABLE 3:** Natural compounds that modulate PCSK9 and lipid metabolism.

Compounds	Biological activity	Methods
Berberine	• Activates AMPK; suppresses SREBP2	In vitro: HepG2 cells [120, 121]
	• Reduces circulating PCSK9 and LDL cholesterol levels	In vivo: mice expressing a luciferase reporter gene [122]
	• Decreases macrophage infiltration and inflammatory markers	
	• Lowers systemic inflammation	

Resveratrol	• Inhibits the SREBP2 pathway	In vitro: human L02 hepatocytes [123]
	• Reduces PCSK9 levels	In vivo: male Wistar rats [124]
		Clinical trial: participants taking high‐dose resveratrol supplements [125].

Quercetin	• Inhibits transcription factor HNF1*α*	In vitro: the mouse IEC line mode‐K [108]
	• Demonstrates PCSK9‐reducing ability in animal models	In vivo: male ApoE−/− mice [107], male and female C57BL/6J mice [106]
	• Improved cardiovascular markers	Clinical trial: participants taking supplemental doses (500–1000 mg/day) [127]

EGCG	• Interfere with the enzymatic maturation of the PCSK9 protein	In vitro: the HepG2 cell line and Huh7 cell line [128].
	• LDL‐lowering effect and plasma PCSK9	In vivo*:* male rats Sprague‐Dawley (SD) [128].
		Clinical trial: participants who consumed one cup (250–300 mL) of green tea daily [128].

Curcumin	• Modulates the IDOL/LDLR pathway	In vitro: HepG2 cells in *vivo*: male Wistar rats [131].
	• Shows positive effects on PCSK9 in animal models	

Brazilin	• Inhibits PCSK9 activity with directly disrupts PCSK9–LDLR interaction at the EGF‐A domain of LDLR	In silico: molecular docking (AutoDock Vina via PyRx 0.98) using PCSK9 crystal structure (PDB ID: 6U26).
		In vitro: IC_50_ ≈ 2.19 μM/0.628 μg/mL [132]

## 7. Clinical Applications and Considerations

The complex interplay among PCSK9 signalling, macrophage polarisation and foam cell formation presents multiple therapeutic opportunities for atherosclerosis intervention. PCSK9 as a key regulator of inflammatory responses and foam cell formation, and the potential of natural products. This section synthesises the translational potential of current findings and outlines promising paths for future research and clinical application.

### 7.1. Integrated Therapeutic Strategies

#### 7.1.1. Dual‐Pathway Targeting

Combining PCSK9 inhibition with modulation of macrophage phenotype offers a promising multitarget approach [90]. While monoclonal antibodies against PCSK9 effectively lower LDL cholesterol, their concurrent anti‐inflammatory effects—mediated through reduced TLR4/NF‐κB Signalling—provide additional vascular protection [10]. Future therapies could enhance this synergy by pairing PCSK9 inhibitors with agents that promote M2 macrophage polarisation or inhibit M1 differentiation.

#### 7.1.2. Natural Compound Formulations

The bioactive compounds discussed in Sections 4–6 present opportunities for novel nutraceutical or pharmaceutical development. Formulations combining curcumin (targeting CD36 and AMPK), fisetin (inhibiting NLRP3) and PCSK9‐modulating compounds like berberine could deliver comprehensive protection against foam cell formation through complementary mechanisms. Such combinations may achieve efficacy at lower individual doses, potentially reducing side effects [133–135].

#### 7.1.3. Personalised Medicine Approaches

Genetic polymorphisms in PCSK9, SR (CD36, LOX‐1) and inflammatory pathway components (TLR4, NLRP3) influence individual susceptibility to atherosclerosis and treatment response. Future strategies could incorporate genetic profiling to identify patients most likely to benefit from specific pathway‐targeted interventions, particularly those involving bioactive compounds with multifaceted mechanisms [44, 136–138].

### 7.2. Mechanistic Insights for Drug Development

#### 7.2.1. PCSK9‐TLR4 Interaction as a Drug Target

The identification of PCSK9 as a TLR4 ligand opens new avenues for small‐molecule development. Compounds that disrupt the PCSK9‐TLR4 interaction without affecting PCSK9’s LDLR regulatory function could offer selective anti‐inflammatory benefits while preserving cholesterol homeostasis. High‐throughput screening platforms leveraging structural details of this interaction could accelerate the discovery of such selective inhibitors [5, 90, 139].

#### 7.2.2. SR Modulation

Rather than complete inhibition, selective modulation of SR activity represents a nuanced therapeutic strategy. Compounds that reduce oxLDL uptake while preserving pathogen clearance functions could be developed by targeting specific receptor domains or associated signalling molecules (e.g., Lyn/Syk kinases in CD36 signalling).

#### 7.2.3. Inflammasome‐Targeted Therapies

The central role of NLRP3 inflammasome activation in PCSK9‐amplified inflammation underscores the therapeutic potential of inflammasome inhibitors. However, careful consideration of timing and specificity is needed, as inflammasome components participate in host defence. Targeted delivery systems (e.g., nanoparticles, macrophage‐specific carriers) could localise inflammasome inhibition to atherosclerotic plaques while sparing systemic immune function.

### 7.3. Biomarker Development and Monitoring

#### 7.3.1. Multiparameter Inflammatory Profiles

Beyond traditional lipid markers, panels incorporating PCSK9 levels, macrophage‐derived EVs, specific cytokine profiles (IL‐1β, IL‐18) [140] and oxidative stress markers could better assess plaque inflammation and treatment response. Such panels would be particularly valuable for monitoring the effects of natural compounds with primarily anti‐inflammatory rather than lipid‐lowering actions.

#### 7.3.2. Imaging Advancements

Novel imaging modalities that specifically detect M1 macrophages, oxidised LDL accumulation or PCSK9 expression within plaques could transform atherosclerosis management. PET tracers targeting TLR4, CD36 or activated NLRP3 could provide real‐time assessment of plaque inflammation and therapeutic response, guiding treatment intensification or modification.

### 7.4. Clinical Translation Challenges and Opportunities

#### 7.4.1. Attenuating Bioavailability

Many promising natural compounds face bioavailability challenges. Advanced delivery systems—including nanoparticle encapsulation, phospholipid complexes and prodrug formulations—could enhance the therapeutic potential of curcumin [141], fisetin [142], Sal B [97] and other bioactive agents. Research into synergistic combinations that improve absorption and tissue distribution is particularly needed.

#### 7.4.2. Standardisation and Quality Control

For natural product‐based therapies, standardisation of active compound concentrations, identification of key synergistic components and quality control across production batches are essential for clinical application [143–145]. Pharmacokinetic and pharmacodynamic studies establishing optimal dosing regimens for atherosclerosis prevention remain limited and represent a critical research gap.

#### 7.4.3. Long‐Term Safety and Efficacy

While natural compounds generally exhibit favourable safety profiles, systematic investigation of long‐term effects—particularly at therapeutic doses and in combination with conventional medications—remains insufficient [145–148]. Large‐scale, long‐term clinical trials are needed to establish cardiovascular event reduction, not merely biomarker improvement, for these promising interventions.

#### 7.4.4. Pharmacokinetic and Translational Limitations of Natural Compounds

Despite the promising anti‐inflammatory and antiatherosclerotic potential of natural compounds targeting PCSK9‐mediated pathways, several translational limitations remain insufficiently addressed [86, 90, 91]. One major challenge is the inherently low oral bioavailability of many polyphenolic compounds, including curcumin, resveratrol, quercetin and berberine [149–151]. These compounds often exhibit poor aqueous solubility, limited intestinal absorption, rapid metabolism and extensive first‐pass hepatic elimination, resulting in low systemic exposure after oral administration. Consequently, the pharmacological concentrations achieved in experimental models may not be readily reproducible in human clinical settings.

In addition to poor absorption, considerable interindividual pharmacokinetic variability may influence therapeutic outcomes. Factors such as age, sex, gut microbiota composition, genetic polymorphisms in metabolic enzymes and transporters, dietary habits and concomitant medications may alter the absorption, distribution, metabolism and elimination of bioactive compounds [152, 153]. Such variability complicates dose optimisation and may contribute to inconsistent efficacy across patient populations.

Another important translational issue concerns dose extrapolation from preclinical studies to humans. Experimental animal studies frequently employ doses substantially higher than those considered practical or safe for long‐term human administration [154, 155]. Although allometric scaling approaches are commonly used, the biological equivalence between animal and human dosing remains uncertain, particularly for compounds with complex metabolism and multiple active metabolites. Therefore, caution is required when interpreting preclinical efficacy data as direct predictors of clinical benefit.

Pharmacodynamic uncertainties also remain a major concern. Many natural compounds exert pleiotropic effects involving multiple signalling pathways beyond PCSK9 inhibition, including the modulation of oxidative stress, inflammatory cytokines, macrophage polarisation, endothelial function and lipid metabolism [134, 156, 157]. While such multitarget activities may provide synergistic therapeutic advantages, they also complicate the identification of primary mechanisms of action and clinically relevant biomarkers. Moreover, the long‐term consequences of chronic modulation of immune‐metabolic pathways remain inadequately characterised.

To address these challenges, future studies should prioritise the development of advanced delivery systems, standardised extract formulations, pharmacokinetic profiling and well‐designed clinical trials incorporating dose‐escalation strategies and biomarker‐guided therapeutic monitoring [158, 159]. Integrating pharmacokinetic–pharmacodynamic modelling with precision medicine approaches may further improve translational reliability and facilitate the clinical development of PCSK9‐targeted natural therapies for atherosclerosis.

## 8. Future Research Priorities and Conclusion

To advance our understanding of PCSK9’s role in atherosclerosis and translate this knowledge into effective therapies, future research should focus on three interconnected core areas:

### 8.1. Deepening Fundamental Mechanisms

More in‐depth exploration of the fundamental biology of PCSK9 is needed, with an emphasis on the following:

a. Signalling Networks: Mapping in greater detail the intracellular signalling cascades influenced by PCSK9, particularly those beyond the known TLR4/NF‐κB pathway. This includes investigating potential interactions with other pattern recognition receptors and core metabolic pathways.
b. Temporal Dynamics: Tracing how PCSK9‐mediated effects change as plaques progress, from early fatty streaks to advanced, vulnerable lesions prone to rupture.
c. Tissue Specificity: Conducting comparative studies to understand PCSK9’s actions in various vascular sites, to explain why susceptibility to atherosclerosis varies across different areas.

### 8.2. Developing More Advanced Preclinical Models

To bridge the gap between basic science and clinical application, more cutting‐edge experimental systems are required, such as developing models that incorporate humanised PCSK9 pathways, specific macrophage reporter systems and accelerated atherosclerosis to make predictions of therapeutic responses in humans more accurate. Organ‐on‐a‐chip platforms can integrate endothelial cells, vascular smooth muscle cells, and macrophages to better reproduce key cellular interactions involved in atherosclerosis. Such platforms can serve as high‐capacity tools for screening compounds and testing their effects on key processes like foam cell formation.

### 8.3. Innovative Clinical Trial Design

To accelerate the assessment of new therapies, clinical trial frameworks need modernisation. Priorities include implementing adaptive platform trials and N‐of‐1 studies to more efficiently test combination therapies, natural compounds and personalised treatment approaches. Moving beyond merely measuring traditional cardiovascular clinical outcomes by routinely incorporating advanced imaging and novel biomarkers as assessment parameters. This will provide a more complete picture of a therapy’s mechanism of action and its impact on atherosclerotic plaque biology.

PCSK9 functions not only as a regulator of LDLR degradation but also as a critical mediator linking lipid dysregulation, oxidative stress, inflammatory signalling and macrophage‐derived foam cell formation during atherosclerosis progression. Given the multifaceted role of PCSK9 in both lipid metabolism and vascular inflammation, natural bioactive compounds targeting PCSK9‐associated inflammatory pathways may represent promising multitarget therapeutic candidates capable of simultaneously modulating lipid homeostasis and inflammatory responses, although further mechanistic validation and large‐scale clinical studies remain necessary.

## Author Contributions

Maharani Hestu Mukti Wisesa and Anisa Qisti Mathriul: conceptualisation and writing–original draft. Vicko Suswidiantoro and Meidi Utami Puteri: writing–review and editing. Mitsuyasu Kato: writing–review and editing and methodology. Muhd Hanis Md Idris: methodology and data curation. Fadlina Chany Saputri: supervised the findings of this work.

## Funding

This study was funded by the Directorate of Research and Development, Universitas Indonesia, under Hibah PUTI 2025 (No. PKS‐52/UN2.RST/HKP.05.00/2025); and The Indonesian Endowment Fund for Education (LPDP) on behalf of the Indonesian Ministry of Higher Education, Science and Technology and managed under the EQUITY Program, Nos. 4302/B3/DT.03.08/2025 and 573/PKS/R/UI/2025.

## Disclosure

All authors have read and agreed to the published version of the manuscript.

## Conflicts of Interest

The authors declare no conflicts of interest.

## Data Availability

Data sharing is not applicable to this article as no datasets were generated or analysed during the current study.
